# Quantitative Assessment of Histone H2B Monoubiquitination in Yeast Using Immunoblotting

**DOI:** 10.3390/mps5050074

**Published:** 2022-09-24

**Authors:** Andrew M. Leng, Kaitlin S. Radmall, Prakash K. Shukla, Mahesh B. Chandrasekharan

**Affiliations:** 1 Department of Radiation Oncology, University of Utah School of Medicine, Salt Lake City, UT 84112, USA; 2 Huntsman Cancer Institute, 2000 Circle of Hope, Salt Lake City, UT 84112, USA

**Keywords:** histone H2B monoubiquitination, histones, histone modification, epigenetics, Western blot, immunoblot, antibodies, yeast

## Abstract

Studies in *Saccharomyces cerevisiae* and *Schizosaccharomyces pombe* have enhanced our understanding of the regulation and functions of histone H2B monoubiquitination (H2Bub1), a key epigenetic marker with important roles in transcription and other processes. The detection of H2Bub1 in yeasts using immunoblotting has been greatly facilitated by the commercial availability of antibodies against yeast histone H2B and the cross-reactivity of an antibody raised against monoubiquitinated human H2BK120. These antibodies have obviated the need to express epitope-tagged histone H2B to detect H2Bub1 in yeasts. Here, we provide a step-by-step protocol and best practices for the quantification of H2Bub1 in yeast systems, from cell extract preparation to immunoblotting using the commercially available antibodies. We demonstrate that the commercial antibodies can effectively and accurately detect H2Bub1 in *S. cerevisiae* and *S. pombe*. Further, we show that the C-terminal epitope-tagging of histone H2B alters the steady-state levels of H2Bub1 in yeast systems. We report a sectioned blot probing approach combined with the serial dilution of protein lysates and the use of reversibly stained proteins as loading controls that together provide a cost-effective and sensitive method for the quantitative evaluation of H2Bub1 in yeast.

## 1. Introduction

Nucleosomes, the basic units of eukaryotic chromatin, contain DNA wrapped around an octamer of histone proteins (two copies of H3, H4, H2A and H2B, or their variants). Post-translational modifications (PTMs) of histones act in nucleosome remodeling and allow access to the DNA template by replication and transcriptional machineries [1,2,3]. The covalent conjugation of the 76 amino-acid ubiquitin, one of the largest PTMs, onto histones H2A and H2B is important for cellular growth and development [4,5]. Histone H2A, referred to as chromosomal protein A24, was in fact the first reported ubiquitinated protein [6,7]. In mammals, the monoubiquitination lysine-119 of histone H2A (H2AK119ub1) and of lysine-120 of histone H2B (H2BK120ub1) play key roles during transcription and other nuclear processes [5,8,9,10,11], and the misregulation of both these ubiquitination events is linked to human diseases [12,13,14,15].

Extensive studies conducted in simple eukaryotic model systems, *Saccharomyces cerevisiae* (budding yeast) and *Schizosaccharomyces pombe* (fission yeast), have enhanced our basic understanding of the regulation and functions of monoubiquitination of histone H2B at K123 (in *S. cerevisiae*) or K119 (in *S. pombe*) (both equivalent to mammalian histone H2B K120). Unlike mammals, these yeasts lack histone H2AK119ub1 but contain histone H2BK123/K119ub1 [16]. Thus, they provide facile yet powerful systems to examine the effects of histone H2B monoubiquitination (H2Bub1) on chromatin structure, function, and transactions. Enzymes and factors involved in regulating H2Bub1 are conserved from yeasts to humans. The histone H2B ubiquitin-conjugating (HUC) complex in *S. cerevisiae* is made up of a single Rad6 E2 enzyme, a homodimer of the Bre1 E3 ligase, and the Lge1 accessory protein [17,18]. The equivalent *S. pombe* HUC complex, termed HULC, contains Rhp6, a heterodimer of the E3 ligases Brl1/Rfp2 and Brl2/Rfp1, and Shf1 [19,20]. Their orthologous mammalian HUC complexes contain one of two Rad6 homologs UBE2A or UBE2B, a heterodimer of the E3 ligases RNF20 and RNF40, and the accessory protein WAC [21,22,23]. Although the SAGA complex-associated Ubp8 appears to be the only enzyme that deubiquitinates H2BK119ub1 in *S. pombe* [24], Ubp8 and Ubp10 deubiquitinate H2BK123ub1 in *S. cerevisiae* [25,26,27]. Multiple deubiquitinases (DUBs), including USP22 in the SAGA complex, remove ubiquitin from H2BK120 in mammals [28]. Thus, the two yeast systems lack the extensive redundancy and complexity of factors involved in regulating H2Bub1 that is seen in mammals.

Higher eukaryotes contain many histone genes arranged in clusters. In contrast, *S. pombe* contains one gene (*htb1+*) and *S. cerevisiae* contains just two genes (*HTB1* and *HTB2*) that code for histone H2B. This means that one can create loss-of-function alleles in these yeast by mutating the site of monoubiquitination in histone H2B (K119R in *S. pombe* or K123R in *S. cerevisiae*). This, along with their short life cycles and the ease of genome manipulation, make the *S. cerevisiae* and *S. pombe* model systems highly suitable for studies of the structure–function relationships and regulatory networks involved in governing H2Bub1.

Immunoblotting, or Western blotting, is a workhorse technique that is widely used to measure the presence and abundance of H2Bub1 in *S. cerevisiae* and *S. pombe*. In these yeast model systems, the presence of ubiquitin conjugated to H2B has been detected using antibodies against epitope tags, such as Flag, fused to the N- or C- terminus of histone H2B [29,30]. However, the commercial availability of antibodies that recognize yeast histone H2B and, more importantly, those that specifically recognize the ubiquitinated form of histone H2B have obviated the need to express epitope-tagged histone H2B for detecting H2Bub1. Moreover, as shown here, C-terminal epitope-tagging of histone H2B obstructs its detection and can alter the steady state levels of H2Bub1. Here, we provide a streamlined step-by-step procedure for the quantitative assessment of H2Bub1 levels in *S. cerevisiae* and *S. pombe* with immunoblotting using the commercially available antibodies against histone H2B and H2Bub1.

## 2. Experimental Design

**Figure 1:**
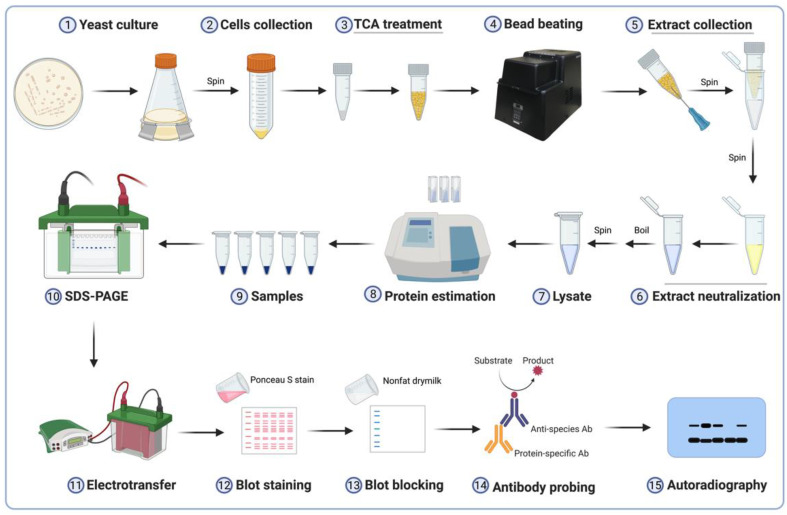
Workflow for the quantitative assessment of histone H2B monoubiquitination using immunoblot.

The workflow for the quantitative assessment of histone H2B monoubiquitination (H2Bub1) in *S. cerevisiae* and *S. pombe* model systems is shown in Figure 1. Yeast cells from a solid media plate are inoculated into and grown overnight in liquid media. These pre-cultures are then used to seed, grow, and harvest mid-log or actively growing yeast cultures (Steps 1–2). Cells are harvested, and then washed and lysed by bead-beating in the presence of trichloroacetic acid (TCA) (Steps 3–4). The acidic cell extract is collected, neutralized with Tris base, and boiled to obtain the final denatured lysate (Steps 5–7). After estimation of protein concentration using spectrophotometry, samples with equal amounts of proteins are resolved by sodium dodecyl sulfate-polyacrylamide gel electrophoresis (SDS-PAGE) and transferred onto a polyvinylidene fluoride (PVDF) membrane using electro-transfer (Steps 8–11). Subsequently, blots are reversibly stained with Ponceau S dye to demarcate the regions containing H2Bub1 or H2B. The blot is blocked with non-fat dry-milk and sequentially probed with an antigen-specific primary antibody and a horse radish peroxidase (HRP)-conjugated species-specific secondary antibody (Steps 12–14). After incubating the antibody-probed blot with the ECL Plus substrate, the ensuing chemiluminescent signal is captured by autoradiography or other imaging methods and quantified by densitometry (Step 15).

### 2.1. Materials

1. Yeast extract (Fisher Scientific, Waltham, MA, USA; Cat. no.: DF0127-08-0)
2. Bacto™ Peptone (Fisher, Waltham, MA, USA; Cat. no.: DF0118)
3. Adenine hemisulfate salt (Sigma, St. Louis, MO, USA; Cat. no.: A9126)
4. D-(+)-Glucose monohydrate (Sigma, St. Louis, MO, USA; Cat. no.: 49159)
5. Bacto™ Dehydrated Agar (Fisher, Waltham, MA, USA Cat. no.: DF0140-01-0)
6. YES50 (Sunrise Science, Knoxville, TN, USA; Cat. no.: 2009-1 kg)
7. Trichloroacetic acid solution (TCA) (Sigma, St. Louis, MO, USA; Cat. no.: T0699-100 mL)
8. Tris (Fischer, Cat. no.: BP152-500 g)
9. 2-Mercaptoethanol (BME) (Sigma, St. Louis, MO, USA; Cat. no.: M3148-100 mL)
10. Bovine Serum Albumin (BSA) (New England Biolabs, Ipswich, NY, USA; Cat. no.: B9000S)
11. ProSignal^®^ full range prestained protein ladder (10-250 kDa) (Genesee Scientific, San Diego, CA, USA; Cat. no.: 83-660)
12. Tween-20 (Sigma, St. Louis, MO, USA; Cat. no.: P9416)
13. 30% Acrylamide/Bis Solution (29:1) (Bio Rad, Hercules, CA, USA; Cat. no.: 1610156)
14. N′-Tetramethylethylenediamine (TEMED) (Bio Rad, Hercules, CA, USA; Cat. no.: 161-0801)
15. Ammonium Persulfate (APS) (Sigma, St. Louis, MO, USA; Cat. no.: 016-060-006)
16. Glycine (Fischer, Waltham, MA, USA; Cat. no.: BP381-5 kg)
17. Sodium Dodecyl Sulfate (SDS) (Sigma, St. Louis, MO, USA; Cat. no.: L3771)
18. PVDF Membranes 0.45 μm, (VWR, Radnor, PA, USA; Cat. no.: 490007-440)
19. Blotting Paper (VWR, Radnor, PA, USA; Cat. no.: 732-0591)
20. Ponceau S (Fisher, Waltham, MA, USA; Cat. No BP103-10)
21. Nestle Carnation Instant Nonfat Dry Milk (Nestle, Arlington, VA, USA)
22. Ampac 500 Series Tubular Rollstock (Thomas Scientific, Swedesboro, NJ, USA; Cat. no.: TRS-95125-3)
23. 2X Laemmli Sample Buffer (Bio Rad, Hercules, CA, USA; Cat. no.: 1610737)
24. Anti-histone H2B (S. pombe) antibody (Gene Tex, Irvine, CA, USA; Cat. no.: GTX64122)
25. Anti-histone H2B antibody (Active Motif, Carlsbad, CA, USA; Cat. no.: 39237)
26. Anti-histone H3 antibody (Epicypher, Durham, NC, USA; Cat. no.: 13-0001)
27. Anti-Ubiquityl-histone H2B (Lys120) antibody (D11) (Cell Signaling Technology, Danvers, MA, USA; Cat. no.: 5546)
28. *DC*™ Protein Assay Kit I (Bio Rad, Hercules, CA, USA; Cat. no.: 5000111)
29. ECL Plus Western Blotting Substrate (Thermo Fisher Scientific, Waltham, MA, USA; Cat. no.: 32312)
30. Apollo Transparency Film (Office Depot, Boca Raton, FL, USA; Model: VPP201CE)
31. Image J software (https://imagej.nih.gov/ij/) (accessed on 29 July 2022)

### 2.2. Equipment

32. 1.5-mL Screw-cap tubes (Genesee Scientific, San Diego, CA, USA; Cat. no.: 21-257)
33. 1.5-mL Microfuge tubes (Genesee Scientific, San Diego, CA, USA; Cat. no.: 14-125)
34. 1.5-mL Snap-cap tubes (Genesee Scientific, San Diego, CA, USA; Cat. no.: 14-214)
35. 16-mL Culture tubes (Genesee Scientific, San Diego, CA, USA; Cat. no.: 21-310)
36. 50-mL Centrifuge tubes (Genesee Scientific, San Diego, CA, USA; Cat. no.: 28-108)
37. Mini Bead Beater 96 (Biospec Products, Bartlesville, OK, USA; Cat. no.: 1001)
38. 0.7-mm Zirconia Beads (Biospec Products, Bartlesville, OK, USA; Cat. no.: 11079107zx)
39. 16-place Beaker Buddy™ (VWR, Radnor, PA, USA; Cat. no.: 100493-762)
40. Corning™ Hot Plate Stirrer (Fisher, Waltham, MA, USA; Cat. no.: 07-770-152)
41. 200-μL Round gel tips, 0.58 mm (Genesee Scientific, San Diego, CA, USA; Cat. no.: 14-100)
42. Eppendorf Centrifuge 5424 (Eppendorf, Hamburg, Germany; Cat. no.: 5424)
43. Allegra 6R Centrifuge (Beckman Coulter, Brea, CA, USA; Cat. no.: BE-A6R)
44. BD Precisionglide^®^ Syringe Needles (18 gauge) (Sigma, St. Louis, MO, USA; Cat. no.: Z118044)
45. Ultrospec 2100 Pro UV/Vis Spectrophotomoter (Biochrom US, Holliston, MA, USA; Cat. no.: 80-2112-21)
46. Humboldt High Temperature Burner (Humboldt Mfg Co. Elgin, IL, USA; Cat. no.: H-5500)
47. Petri plates (Falcon, Corning, NY, USA, Cat. no.: 351-029)
48. Benchtop Electrophoresis Station (CBS Scientific, San Diego, CA, USA; Cat. no.: MGV-202-20)
49. Trans-Blot Cell (Bio Rad, Hercules, CA, USA; Cat. no.: 1703939)
50. PowerPac Basic (Bio Rad, Hercules, CA, USA; Cat. no.: 1645050)
51. ENDURO™ 300 V Power Supply (Labnet, Edison, NJ, USA; Cat. no.: E0303)
52. GyroMini™ Nutating Mixer (Labnet, Edison, NJ, USA; Cat. no.: S0500)
53. Laboratory Platform Rocker (Genesee Scientific, San Diego, CA, USA; Cat. no.: 33-208DXL)
54. X-ray Film Cassette (Thomas Scientific, Swedesboro, NJ, USA; Cat. no.: 1139A30)
55. Epson Perfection V39 Scanner (Epson, Los Alamitos, CA, USA; Model: B11B232201)

## 3. Procedure

### 3.1. Yeast Culture and Harvest of Cells

(1) Streak yeast strains onto YPAD or YES50 solid media (see Section 5: Reagents setup) from glycerol stocks stored at −80 °C. Grow yeast in an incubator for 2–3 days at appropriate temperature.
(2) Use aseptic conditions to inoculate 2–3 yeast colonies into 3–5 mL YPAD or YES50 liquid media in a 16-mL culture tube. Grow cultures at 30 °C or at a permissive temperature with vigorous agitation overnight.
**Technical notes:** *(a)* In general, *S. cerevisiae* or *S. pombe* strains are grown at 30 °C. However, optimal growth for certain *S. pombe* strains including those used in this study, is between 25 °C to 29 °C or at 32 °C. Any conditional temperature-sensitive yeast mutant should be grown at a predetermined permissive or restrictive temperature depending on the experiment. *(b)* We have observed higher levels of H2Bub1 in yeast cells grown in rich media (YPAD or YES50) than in synthetic media. *(c)* To ensure effective aeration, place the culture tubes in a tube holder angled at ~45° in a shaker incubator or, alternatively, grow yeast cultures in baffled Erlenmeyer flasks.
**CRITICAL STEP** In *S. cerevisiae*, the steady-state H2Bub1 levels are dependent on glucose levels [29,31]. Thus, to prevent caramelization of glucose, avoid prolonged exposure of yeast media or stock solutions with glucose to temperatures above 120 °C as used during autoclaving. Alternatively, yeast media with glucose should be prepared by addition of a filter-sterilized glucose stock solution to autoclaved media.
(3) Determine absorbance at 600 nm (OD_600_) of the yeast cultures using a spectrophotometer. To ensure samples are in a linear range of detection, dilute the overnight cultures in growth media and mix well prior to spectrophotometry.
**CRITICAL STEP** The levels of H2Bub1 are high in actively growing cultures and decrease in stationary phase [29]; therefore, cultures should not be overgrown. When analyzing H2Bub1 levels, harvest cells at OD600 0.8–1.0 (or at mid-log phase).
(4) Inoculate yeast cultures at a starting OD_600_ of 0.2 in 50 mL media in a 250 mL baffled flask. Grow with agitation at 30 °C or at an appropriate temperature till cultures reach an OD_600_ between 0.8–1.0; this should take ~4 h.
**Technical notes**: *(a*) Baffled flasks enable better growth by disrupting cell aggregation. *(b)* For effective aeration of yeast cultures, use a media volume that is equal to one-fourth the total indicated capacity of the flask.
(5) Harvest cells by centrifugation (845× *g*) in a 50-mL centrifuge tubes for 3 min.
**Technical notes**: A 50-mL culture grown to 1 OD_600_ yields ~1 × 10^7^ yeast cells. In general, 5-30 ODs of cells are sufficient for the TCA lysis protocol described below with 12–20 ODs being ideal for effective lysis. Using >30 ODs results in poor lysis during bead beating and therefore low protein yields.
(6) Discard the spent media after centrifugation. Resuspend the pellet (5–30 ODs) in 1 mL sterile water to transfer the cells into a labeled 1.5-mL screwcap tube. Centrifuge at 845× *g* for 3 min to pellet cells. Discard water by pipetting or by using vacuum suction.
(7) Resuspend cells in 0.5 mL 5% TCA. Vortex. Centrifuge at 845× *g* for 3 min. Discard the TCA solution and store the cell pellet at −80 °C.
**Technical note**: The cell pellet obtained washing with water will be straw colored, it will be white after washing with 5% TCA.
**PAUSE STEP** The cell pellets can be stored indefinitely at –80 °C.

### 3.2. Lysate Preparation

**Figure 2:**
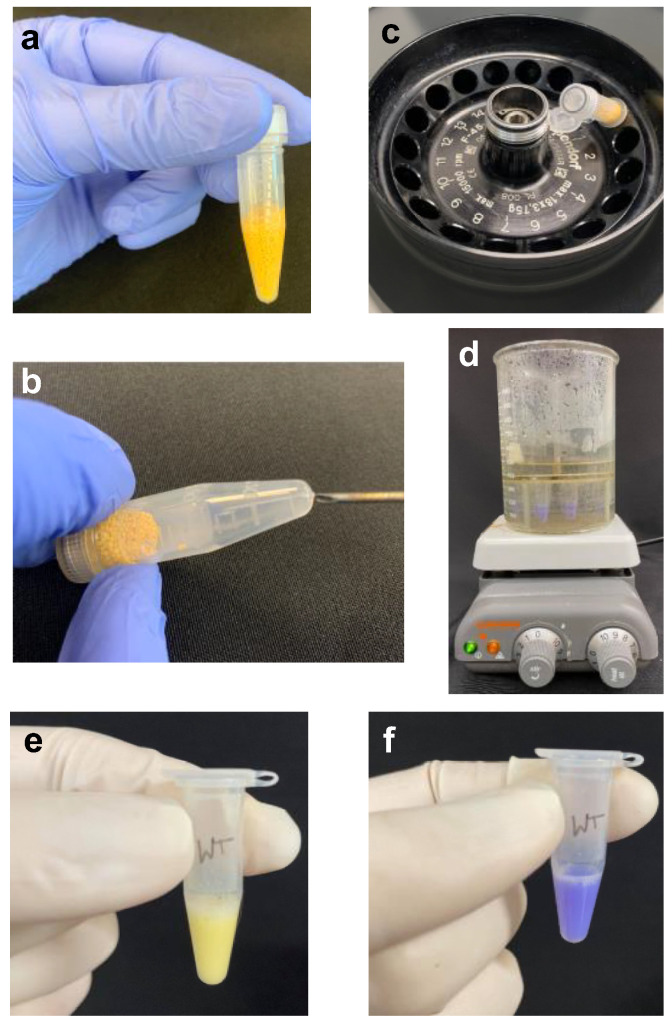
Steps for collection and neutralization of acidic yeast lysate. (**a**). Zirconia beads are added up to the 500 μL mark in a screwcap tube as shown. (**b**). Use a heated needle to puncture the tube at the bottom. (**c**). Use a rotor that will accommodate the screwcap tube-collection tube set up as shown. To prevent it from breaking during centrifugation, the cap of the collection tube is oriented below in the rotor as shown. (**d**). The set up needed for sample boiling is shown. Ensure that the water level is below the tube caps. (**e**). Color of the acidic lysate. (**f**). Color of the lysate after neutralization with Tris base.

(1) Add 0.5 mL 20% TCA to the frozen cell pellet. Thaw and resuspend the cells in the TCA solution by vigorous vortexing.
(2) Add zirconia beads to the 0.5 mL mark of a screwcap tube containing cells resuspended in TCA (Figure 2a). Secure the cap well and place the tube in a Mini-Beadbeater-96. Agitate to bead-beat the cells for a duration of 30 s three times with a short cooling period (5–10 s) between each agitation.

**CRITICAL STEPS***(a)* Do not add more zirconia beads than recommended, as an excess will impede effective cell lysis. *(b)* Place the tubes in the bead-beater such that the tube-restrainer lid can be evenly secured using the wing nut screws. Skewed or imbalanced placement of tubes in the bead-beater can lead to loosening of the screws and dislodging of the lid during the vigorous shaking or bead-beating step that can potentially damage the equipment or the sample tubes.
(3) Heat the end of an 18-gauge needle using a Bunsen burner and insert it into the bottom of the screwcap tube (Figure 2b).
**CAUTION** Take care not to impale your fingers with the heated needle.
(4) Place the punctured tube in a labeled 1.5-mL microfuge tube; the latter will serve as the collection tube. Centrifuge briefly (5–10 s) to collect the sample (Figure 2c).
**CAUTION** To avoid breaking the sample tube, confirm that sufficient clearance exists between the screwcap-collection tube and the centrifuge lid (Figure 2c).
(5) Discard the screw-cap tube containing beads. Centrifuge the lysate in the collection tube at 845× *g* for 5 min.
(6) Add 300 mL water to a 500-mL glass beaker; bring to boiling.
**Technical note:** This preparative step enables quick processing of samples in step 10.
(7) Discard the supernatant. Wash the pellet gently with 0.5 mL 5% TCA. Centrifuge briefly and discard residual TCA solution.
**CRITICAL STEP** Completely remove all TCA solution, as it will interfere with effective neutralization of the lysate in step 9.
(8) Add 0.3 mL 1X SDS lysis buffer (without reducing agent) to the pellet and resuspend it thoroughly by pipetting and vortexing.
**Technical note:** The bromophenol blue dye in the SDS lysis buffer will turn yellow upon addition to the acidic sample (Figure 2d).
(9) Neutralize the lysate by addition of 5–10 μL of the 2 M Tris base at a time and vortexing. Continue to add base until the sample turns blue (Figure 2e).
**Technical note:** Approximately 20–50 μL 2M Tris base will be needed to neutralize 300 μL of the acidic lysate.
(10) Securely fasten the tubes in a tube holder and place in the boiling water bath (step 6) for 8 min.
**Technical note:** The water level in the beaker should be below the tube caps to prevent seepage and dilution of samples (Figure 2f).
**CAUTION** Exercise care to avoid scalding or burns when handling the heated hot plate and the boiling water bath.
(11) Centrifuge the lysate at 16,363× *g* for 10 min at room temperature.
(12) Transfer the supernatant to a 1.5-mL microfuge tube.
**PAUSE STEP** Samples can be stored indefinitely at −80 °C.

### 3.3. Estimation of Protein Concentration

**Figure 3:**
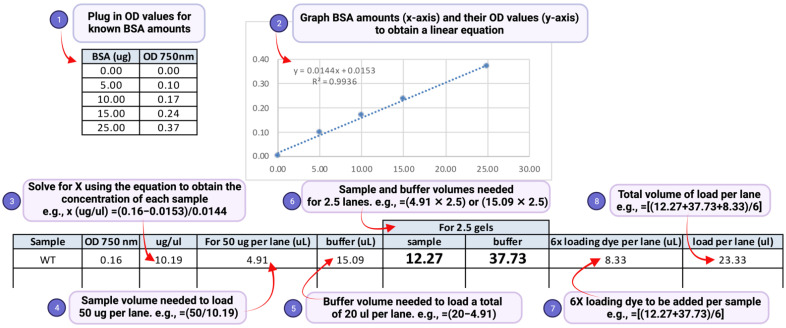
Steps to calculate protein concentration of the cell lysate using a spreadsheet.

(1) As samples are prepared using a buffer containing SDS, protein estimation should be performed using the detergent-compatible Bio-Rad *Dc* assay. Add 5 μL of sample to 20 μL water in a 1.5-mL microfuge tubes. Mix well by pipetting and transfer 5 μL of the diluted sample to 20 μL water in another 1.5-mL microfuge tube.
**Technical note:** If using samples stored at −80 °C (Section 3.2, step 10), thaw samples in a boiling water bath or heat block set >95 °C, then vortex well and centrifuge at 16,363× *g* at room temperature for 5 min to remove any insoluble particulates or precipitates. Transfer the lysate to a fresh labeled tube before proceeding to protein estimation.
(2) To prepare a reference or protein standard for spectrophotometry, add 10 μL of 20 mg/mL BSA stock to 90 μL water to obtain a 1 μg/μL working solution. Label 6 tubes as follows: 0, 0, 5, 10, 15 and 25. Add 25 μL water to the tubes labeled ‘0′; this will serve as a blank and as a ‘no BSA’ control. To the tubes labeled 5, 10, 15 and 25, add 5 μL 1 μg/μL BSA and 20 μL water, 10 μL 1 μg/μL BSA and 15 μL water, 15 μL 1 μg/μL BSA and 10 μL water, and 25 μL 1 μg/μL BSA, respectively.
(3) Prepare the *Dc* assay reagent mix as follows: Add 20 μL of reagent S to 1 mL of reagent A to obtain reagent SA. Add 125 μL reagent SA to 25 μL sample and to each BSA standard and then add 1 mL of reagent B to each sample and standard. Mix well and incubate at room temperature for 15 min. Transfer the reaction mix into a cuvette and determine absorbance at 750 nm using a spectrophotometer.
(4) Transfer the recorded absorbance values to a Microsoft Excel spreadsheet. Compute the protein concentration of the samples and amounts of sample to be used in gel electrophoresis as shown in Figure 3.

**Technical notes:***(a)* An aliquot of 50 μg of the total denatured lysate is in general sufficient for detection of H2Bub1. *(b)* A two-fold serially diluted gradient of protein amounts (e.g., 50 μg, 25 μg, and 12.5 μg) should be used to ensure that the signal is in the linear range of detection. *(c)* Load the same volume for all samples in each gel lane by diluting to an equal volume with SDS lysis buffer (Figure 3, step 5). This will ensure that the samples migrate uniformly in the gel, and the bands for H2Bub1 or H2B are not stretched or compressed after immunoblotting, *(d)* Prepare two separate sets or aliquots of the samples. The second set can be used in case a repeat run is needed due to poor transfer of proteins onto the blot or due to other problems during electrophoresis or immunoblotting. *(e)* To account for possible sample loss during the boiling or pipetting steps, prepare more than will be needed. For example, as shown in Figure 3 step 6, if a sample is to be loaded in two gel lanes, prepare enough for loading 2.5 lanes. *(f)* In the event that samples must reanalyzed, thaw the frozen lysate quickly in a heat block set at >95 °C. Vortex and spin to remove any precipitates. Measure the protein concentration again using the *DC* assay and recalculate concentration and volumes needed for preparation of samples for electrophoresis.

### 3.4. Electrophoretic Separation of Proteins

(1) Set up the gel casting/running unit by assembling the cleaned gel plates along with spacers. Pipet in the gel solution containing 15% acrylamide: bis-acrylamide and SDS. Expedite polymerization by applying isopropanol or ethanol on top of the gel mix to limit exposure to air. After polymerization, discard isopropanol or ethanol, wash it away with water, remove any residual water with a blotting paper. Add the mixture for the stacking gel and insert the comb.
(2) After polymerization, remove the spacer or gasket, if any, at the bottom of the gel plates. Fix the gel onto an electrophoresis apparatus with clips. Fill the top and bottom tanks of the rig with the SDS running buffer. Remove the comb and flush the wells with the running buffer using an 18-gauge needle fixed to a syringe to remove any gel debris. Use a syringe with a bent needle to flush the bottom of the gel to remove any trapped air bubbles.
(3) Mix SDS loading buffer and sample in amount calculated as describe above (Figure 3, step 7). Mix well and boil for 8 min.
(4) Briefly centrifuge to return any condensate to solution and load equal volumes of the denatured samples into individual wells. Load a lane with the protein ladder.
**Technical notes:** To ensure that samples run evenly in the gel, and that the bands are not ‘squeezed’ or ‘smiling’ after electrophoresis, the volumes of all samples, including the protein ladder, should be the same. Fill any empty lanes with ‘mock’ samples, which are made up of the lysis buffer and the SDS sample buffer in the same volume as the samples.
(5) Electrophorese at 75 V until the dye front enters the separating gel, then increase to 130–140 V and run for 3–4 h until the 10-kDa size marker reaches the bottom of the gel.

### 3.5. Immunoblot for H2Bub1

**Figure 4:**
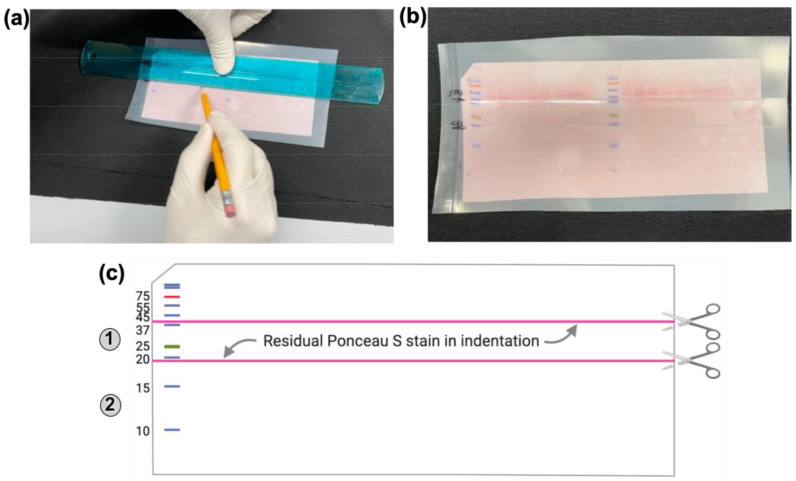
Steps involved in Ponceau S staining and sectioning of the membrane. (**a**). The blot is placed in a plastic pouch and sections of the Ponceau S-stained to be cut are marked with a pencil. (**b**). The blot with marked sections before blocking are shown. (**c**). After blocking, the Ponceau S stain lodges into the indentation made by the Pencil mark and acts as a guide for cutting the blot into sections for probing with antibodies to assess H2Bub1 (1) or H2B (2) levels.

(1) Cut four blotting papers and a PVDF membrane to fit the dimensions of the gel. Wet and activate the PVDF membrane in methanol and then place it in transfer buffer.
**CRITICAL STEP** It is important to cut the blotting papers and the PVDF membrane to exactly fit the dimensions of the gel in order to prevent trapping of any air bubbles, which can block the transfer of proteins from the gel onto the membrane.
(2) Disassemble the gel apparatus and remove the gel from the glass plates. Remove the wells and/or the stacking gel and equilibrate the gel in transfer buffer.
(3) In the gel holder cassette, place one fiber pad or sponge prewetted with transfer buffer, two pre-cut, pre-wetted blotting papers, the equilibrated gel, the activated PVDF membrane, the final two additional pre-cut, pre-wetted blotting papers, and the second fiber pad.
**Technical note**: Remove any trapped air bubbles using a gel roller or by gently rolling over the gel-blotting paper sandwich with a plastic pipette.
(4) Close the cassette taking care not to cause any shifts in the gel-blotting paper sandwich. Secure the cassette. Place the cassette in the transfer module. Fill the module with cold Towbin transfer buffer (see Section 5). Add a stir bar to maintain even buffer and ion distribution in the tank.
(5) Put the lid on the transfer module ensuring that the plugs are in contact with the correct electrodes. Connect the cables to a power supply and run at constant current (150 mA) for 6 h.
(6) Post transfer, disassemble the gel- blotting paper-fiber pad sandwich. While the membrane with transferred proteins is still wet and on the blotting papers, mark each protein molecular weight marker by placing a dot on the membrane with a lab marker. Clip the left corner of the blot to indicate the protein-containing surface and the loading order of the samples (Figure 4). Label the membrane below the clipped notch with the name of the primary antibody to be used. Then, place the blot in a tray using forceps. Add Ponceau S stain to cover the membrane and stain by rocking in a shaker for 5 min.

(7) Carefully pour the Ponceau S stain back into its storage container. Pour distilled water into a corner of the tray and then rock the tray back and forth to destain the blot evenly. Avoid pouring water directly onto the membrane. Discard water and wash once with water.
**Technical notes:** *(a)* Use forceps to handle the membrane. *(b)* Protein bands should be visible upon Ponceau S staining and can be used to determine the electro-transfer efficiency (Figure 4a,b).
(8) With the stained proteins still visible, use forceps to place the membrane into a plastic bag and seal two sides using a heat sealer. Use a ruler and a pencil to mark the locations to be cut before probing with a primary antibody (Figure 4a,b).
**Technical note:** The Ponceau S stain that remains after washing with water will settle into the indentation made by the pencil marking and this will indicate where the membrane should be cut into sections before probing with primary antibodies (Figure 4c).
(9) Add 10–15 mL of 5% non-fat dry milk (NFDM) prepared in 1X phosphate buffered saline (PBS) to block the membrane to be probed with anti-histone H2B antibody. Use 5% non-fat dry milk prepared in 1XPBS containing 0.05% Tween-20 (PBST) as the blocking agent for all other antibodies. Seal the bag with a heat sealer. Place on a nutating mixer and agitate for 1 h at room temperature.
(10) During the blocking step, prepare primary antibody in 2.5% or 5% non-fat dry milk in 1X PBST. Antibody dilutions used for experiments described here are listed in Table 1.

**Table 1:** Antibody dilutions used.

Antibody	Dilution
Anti-Ubiquityl-histone H2B (Lys120) antibody (D11)	1:1000
Anti-histone H2B antibody (Active Motif)	1:1000 (for 20–35 kDa section)
Anti-histone H2B antibody (Active Motif)	1:10,000 (for 10–20 kDa section)
Anti-histone H2B antibody (Active Motif)	1:1000 (for whole blot)
Anti-histone H2B antibody (GeneTex)	1:1000 (for 20–35 kDa section)
Anti-histone H2B antibody (GeneTex)	1:5000 (for 10–20 kDa section)
Anti-histone H3 antibody	1:5000

(11) Remove the membrane and cut along the marks made in step 8 (Figure 4c). Place each portion in separate plastic bag. Seal the bag on two sides.
(12) Add the primary antibody containing solution and seal the bag. Place on a nutating mixer and agitate at room temperature for 1 h.
**Technical note:** Add the primary antibody dilution for different sections of the membrane as indicated in Table 1.
(13) Place each membrane in a tray. Add 1X PBST and agitate for 15 min at room temperature in an orbital shaker. Repeat the wash step with fresh 1X PBST.
(14) Prepare secondary antibody in 2.5% non-fat dry milk in 1X PBST.
(15) Place each portion of the membrane in a separate bag and seal two ends of each bag. Add the secondary antibody containing solution, seal, and agitate at room temperature for 1 h on a nutating mixer.
(16) Wash the membranes twice in 1X PBST as described in step 13.
(17) During last 5 min of the second washing step, prepare the ECL solution. For every 1 mL of Solution A, add 25 μL Solution B. Mix well and place in dark. Set up a clean glass plate. Place paper towels adjacent to two sides of the glass plate.
(18) After washing, take the blot with a forceps and remove excess wash buffer by dabbing on a paper towel. Place the blot on the glass plate with the protein containing surface facing upwards.
**CRITICAL STEP** Do not let the PVDF membrane dry out.
(19) Add ECL solution dropwise to cover the blot completely. Incubate at room temperature for 5 min. During the last 2 min of incubation, flip the membrane with the protein surface facing downwards to ensure even contact with the ECL solution.
**Technical Notes:** Perform incubation of the blot and the ECL substrate on an even surface, such as, a glass plate. It is easy to add and evenly distribute the ECL substrate solution when the blot is moist.
(20) During incubation, fold a transparency printer film/sheet and cut it to a size slightly larger than the autoradiography film. Tape the two cut pieces of the sheet together along one side.
(21) Lift the membrane with forceps and remove excess ECL solution by draining it onto a paper towel. Place the blot between the two pieces of sheet protector. Place this sandwich in a film cassette.
(22) Expose the blot to an X-ray film in a dark room for different time periods. Develop the films. Perform an overnight or long exposure to visualize the entire blot. Align the film with the membrane and mark locations of the different protein size markers on the film.
**Caution:** *(a)* Use a chemical resistant and smudge-proof lab marker on the PVDF membrane. *(b)* Label and store the membranes safely to be used in subsequent analyses (Section 3.6).

### 3.6. Quantitation of H2Bub1

(1) To account for any discrepancies in the sample amount applied in each gel lane, the signal intensity obtained for any band in the immunoblot must be normalized to the levels of Ponceau S-stained protein bands in the same membrane. To restain the blot with Ponceau S, wet the dry blot initially in transfer buffer and then wash twice with 1X PBST. Add Ponceau S stain to cover the blot and stain by rocking for 5 min at room temperature in a tabletop orbital or rotatory shaker. Pour the stain back in its storage container and then wash the blot evenly with water until protein bands appear to be uniformly stained. Dry the blot in a hood on paper towel then place it with the protein side facing downwards on a flatbed scanner.
**Technical Note:** As the blots are blocked with non-fat dry milk, the background Ponceau S stain on the blot will be higher that obtained after the initial staining performed in Section 3.5.
(2) Choose a developed X-ray film that by visual inspection appears to have signal intensities in a linear range of detection.
**Technical Note:** The linear range of detection is a region where the band intensity on a developed X-ray film is proportional to the amount of target protein in the membrane. Therefore, an X-ray film that shows a gradual decrease in signals commensurate with the serial dilution of extract can be tentatively deemed as being in the linear range of detection and chosen for scanning and quantitation by densitometry. For this initial assessment of signal intensity, it is important to place the developed X-ray film on a piece of white paper to simulate the final scanned image to be used in densitometry.
(3) Scan in gray scale and save the developed X-ray film and the Ponceau S-stained dry blot.
(4) If the scanned image is in .jpg or .png format, open it in Adobe Photoshop and invert the image, so that the bands appear white, and the background appears black. Save the file in .tif format.
(5) Open the .tif file in the Image J software. Select the protein band of interest using the *Rectangle* option. Go to ‘Analyze ‘dropdown menu and use the ‘Measure’ (Cmd M) option to obtain the signal intensity value (Mean). Signal intensities of other protein band(s) are then determined by moving the same rectangle area marker sequentially to select and measure each band. Finally, move the area marker to a section of the image without any band to obtain the ‘background’ signal intensity.
**Critical step:** Use a rectangle of the same size to quantify the bands for a given protein in different lanes to ensure that the area measured remains the same.
(6) Transfer values into an Excel spreadsheet. Subtract the value for the ‘background’ signal from those obtained for the bands of interest. Divide the values obtained for each protein band in the X-ray film with the value obtained for the protein bands in the corresponding lane in the Ponceau S-stained blot. Use these normalized values for plotting graphs using Microsoft Excel or GraphPad Prism.

## 4. Expected Results

**Figure 5:**
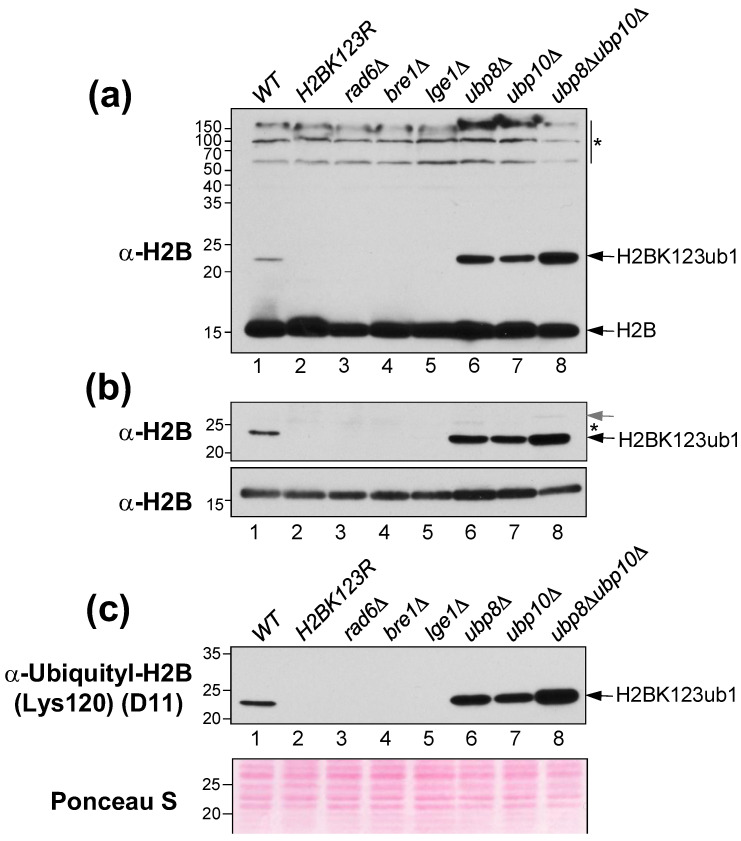
Detection of H2BK123ub1 in the indicated wild-type (WT) and mutant *S. cerevisiae* strains.(**a**) Entire membrane probed with anti-yeast H2B (Active Motif) antibody (1:1000 dilution). (**b**) Sections of membrane probed with anti-yeast H2B (Active Motif) antibody at 1:1000 dilution (upper blot) and 1:10,000 (lower blot). *Black arrow*, H2BK123 monoubiquitinated form. Grey arrow, diubiquitinated form of H2B. (**c**) Section of membrane probed with anti-ubiquityl-H2B (K120) (Cell Signaling Technology) antibody (1:1000). Proteins stained with Ponceau S serve as loading controls. Molecular weights (kDa) based on protein size makers are indicated to the left of each blot. Asterisk, unspecific cross-reacting protein.

Ubiquitination is a labile modification that is rapidly removed by the action of deubiquitinases (DUBs) [32]. Thus, to enable quantification of H2Bub1, yeast cell lysates are prepared using denaturants, such as trichloroacetic acid (TCA), to inactivate the DUBs [33]. As antibodies raised against yeast histone H2B are commercially available, we performed immunoblotting to examine ubiquitination of endogenous histone H2B in cell lysates prepared from mutant *S. cerevisiae* strains with deregulated H2BK123ub1 (Table S1). H2BK123ub1 was absent in budding yeast mutants lacking one of Rad6, Bre1 or Lge1, which are subunits of the histone H2B ubiquitin-conjugating (HUC) complex, and in a strain with a mutation at the site of monoubiquitination on histone H2B (H2BK123R) (Figure 5a, lanes 2–5). In contrast, H2BK123ub1 levels were increased in null mutants lacking the DUBs Ubp8 and/or Ubp10 (Figure 5a, lanes 6–8). These results obtained using the anti-yeast histone H2B antibody match well with those reported previously using epitope tag-specific antibodies [26,32,34]. Thus, the anti-yeast histone H2B antibody eliminates the need to epitope-tag histone H2B, as it effectively detects histone H2B monoubiquitination in *S. cerevisiae* acid extracts.

The anti-yeast histone H2B antibody used at 1:1000 dilution provides a very robust signal that appears to be in a non-linear range of detection (Figure 5a, top panel). Additionally, the high abundance of histone H2B seems to compromise the signal for H2BK123ub1 in the wild-type strain (Figure 5a, top panel), probably by ‘soaking up’ or sequestering the antibody. Furthermore, the anti-yeast histone H2B antibody had cross-reactivity with high-molecular-weight yeast proteins (Figure 5a). To overcome these drawbacks, we probed sections of the membrane with different amounts of the anti-yeast histone H2B antibody (Table 1). When we probed the section of the membrane that contains proteins between 20 and 35 kDa with a 1:1000 dilution antibody, we observed a stronger signal for H2BK123ub1 in the wild-type strain than when probing the whole blot (Figure 5a,b, compare lanes 1). Upon probing the sectioned blot, we also detected the reported diubiquitinated species of histone H2B in the *ubp8Δubp10Δ* double mutant strain [27] (Figure 5b). We probed the lower section of the membrane that contains proteins between 10–20 kDa with a 1:10,000 dilution of antibody. This enabled us to detect the previously reported decrease in histone H2B levels in the *ubp8Δubp10Δ* mutant strain [27,32] (Figure 5a,b). Taken together, these results demonstrate that our sectioned blot probing technique offers higher sensitivity than the probing of the entire membrane and provides signals in a linear range of detection.

Antibodies that specifically recognize histone H2B K120 or K123 ubiquitination in mammalian or yeast systems, respectively, are commercially available. Using the anti-histone H2BK120ub1 antibody (clone D11, Cell Signaling Technology), we obtained robust signals in denatured *S. cerevisiae* extracts (Figure 5c). This antibody was raised against a synthetic branched peptide corresponding to the K120-ubiquitinated form of human histone H2B. That it recognized the *S. cerevisiae* H2BK123ub1 is consistent with prior reports [35,36]. Collectively, our results show that both anti-yeast histone H2B and anti-histone H2BK120 ubiquityl antibodies can effectively detect H2BK123ub1 in denatured *S. cerevisiae* extracts. Compared to the commonly employed full blot probing method, our sectioned blot probing approach when applied with these antibodies provides a cost-effective advantage, as one can use lesser amount of the antibodies and the chemiluminescent detection reagents. Additionally, our approach has a technical advantageous, as sections of the membrane can be used to probe for histone H2B or other yeast proteins and used for Ponceau S dye staining. Thus once can obtain information on multiple factors from a single experiment.

**Figure 6:**
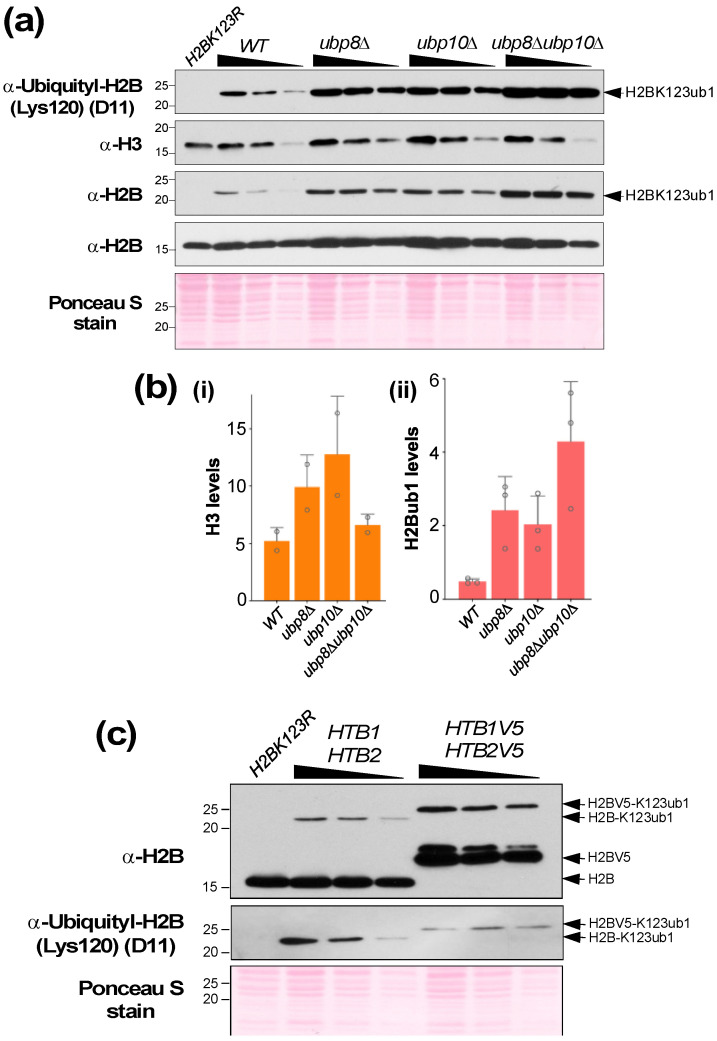
(**a**) Sections of membrane probed with the indicated antibodies to quantify histones H3 or H2B and H2BK123ub1 levels in wild-type (WT) and indicated deubiquitinase mutant S. cerevisiae strains. The antibody dilutions used are listed in Table 1. (**b**) Quantitation of histones H3 (i) and H2BK123ub1 (ii) levels normalized to Ponceau S stained proteins from two or three independent experiments, respectively. (**c**) Sections of membrane probed with anti-H2B antibody (1:1000 dilution) (upper), anti-ubiquityl H2BK120 antibody (1:1000) (middle), and stained with Ponceau S (lower) using extracts prepared from *S. cerevisiae* strains expressing untagged or C-terminal V5 epitope-tagged histone H2B.

**Figure 7:**
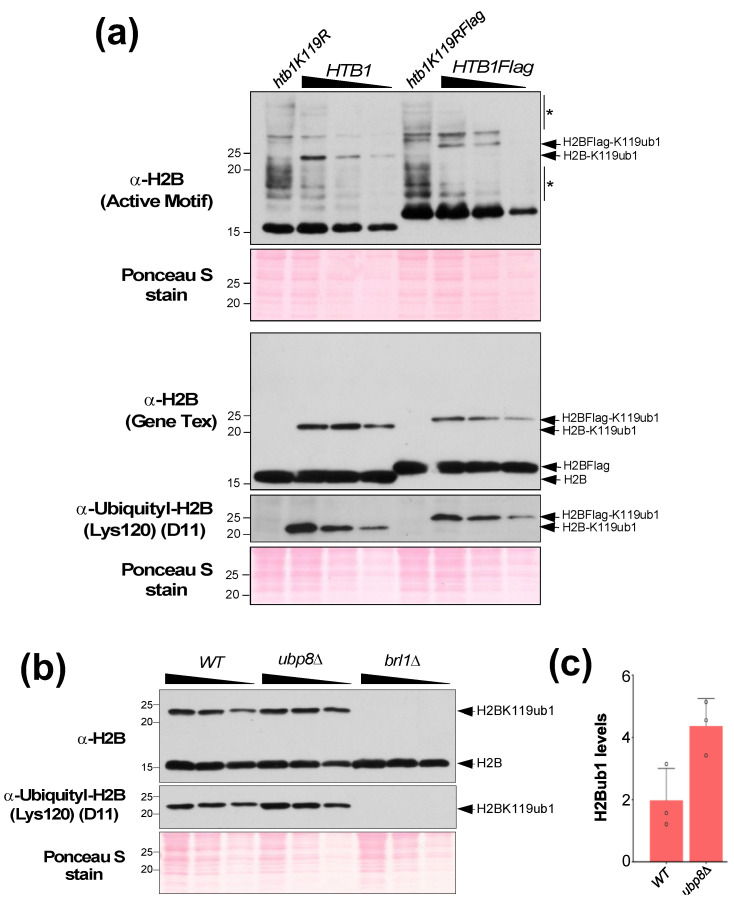
(**a**) Samples from extracts prepared from S. pombe strains expressing untagged H2B or Flag-tagged H2B were spearated and transferred onto a PVDF membrane. Shown are whole or sections of membrane probed with anti-histone H2B antibody from Active Motif and from Gene Tex and with anti-H2BK120 ubiquityl antibody. Ponceau S dye-stained sections used for normalization are also shown. Asterisk indicates cross-reacting proteins. (**b**) Samples from extracts prepared from wild-type S. pombe or strains lacking Ubp8 or Brl1 were separated and transferred to PVDF membrane. Shown are sections of membrane probed with anti-H2B (Gene Tex) or anti-H2BK120 ubiquityl antibody are shown. A section of the membrane stained with Ponceau S used for normalization is also shown. (**c**) Quantitation of H2BK119ub1 levels in the S. pombe ubp8-null mutant relative to wild-type (WT) strain. Error bars denote standard deviation of the mean from three independent experiments.

We also tested the ability of these commercial antibodies to detect histone H2BK119 monoubiquitination (H2BK119ub1) in denatured cell lysates prepared from *S. pombe* strains. Unlike with the *S. cerevisiae* extracts (Figure 5 and Figure 6), the anti-yeast histone H2B antibody (Active Motif; 1:1000) yielded high background or cross-reactivity when applied to the *S. pombe* extracts (Figure 7a, first panel). On the other hand, the anti-histone H2B (*S. pombe*) antibody from Gene Tex provided a ‘clean’ result, displaying a highly specific signal without any cross-reactivity (Figure 7a, third panel). Next, we tested the anti-histone H2BK120 ubiquityl antibody, which detected H2BK119ub1 in denatured cell lysates prepared from *S. pombe* strains (Figure 7a,b). Overall, we find that the anti-human ubiquityl H2BK120 antibody can detect monoubiquitinated histone H2B in both *S. cerevisiae* and *S. pombe* systems.

In our studies with *S. pombe*, we noticed that the H2BK119ub1 levels were reduced in a strain that expresses a C-terminal Flag epitope-tagged histone H2B (HTB1flag) when compared to a strain with untagged histone H2B (HTB1) (Figure 7a). We also observed that the C-terminal epitope-tagging of histone H2B in *S. cerevisiae* caused an increase in H2BK123ub1 levels when compared to the control strain (Figure 6c). Surprisingly, probing with the anti-histone H2BK120 ubiquityl antibody showed that H2BK123ub1 levels were reduced in the same strain (Figure 6c), indicating that the C-terminal epitope-tagging of histone H2B impedes antigen recognition by the antibody. Therefore, these results reveal the drawbacks of C-terminal epitope tagging of histone H2B in budding as well as fission yeast model systems. Overall, our results shown in Figure 5, Figure 6 and Figure 7 together demonstrate the benefits of using antibodies that directly recognize histone H2B or its monoubiquitinated form in the yeast model systems.

To quantitate changes in histone H2B ubiquitination levels, it is important that the signals obtained are in a linear range of detection. Hence, we analyzed a different protein amounts via a two-fold serial dilution of the cell extracts. After gel electrophoresis and electro-transfer, the membrane was sectioned and probed with anti-yeast histone H2B or anti-histone H2BK120 ubiquityl antibody. To account for any discrepancies in protein amounts loaded in gel lanes, the signal for H2Bub1 must be normalized to a loading control in the same sample. As the core constituent of the nucleosome, histone H3 levels are a good loading controls for quantifying histone modifications. However, immunoblotting showed that histone H3 levels were altered in strains deleted for Ubp8 and/or Ubp10 (Figure 6a). Moreover, gene expression analyses also showed that transcripts for various histone genes are altered in yeast mutants lacking the enzymes involved in either conjugating or removing H2BK123ub1 when compared to the control wild-type strain [Chandrasekharan, unpublished observations]. For these reasons, we chose to normalize the signal for H2BK123ub1 from the anti-histone H2BK120 ubiquityl antibody to signals from multiple yeast proteins stained with the Ponceau S dye during densitometry. Our quantitative analyses showed that H2BK123ub1 levels were increased 5–12-fold in the mutant strains (Figure 6a,b), which is in agreement with a previous report [27]. Quantitation of H2BK119ub1 levels in *S. pombe* strains using signals from the Ponceaus S-stained blot for normalization showed that H2BK119ub1 levels were increased approximately 6-fold in the *ubp8Δ* null mutant compared to the wild-type strain (Figure 7c), which is similar to the increased observed in the *S. cerevisiae ubp8Δ* null mutant (Figure 6a). Overall, these results demonstrate that the anti-histone H2BK120 ubiquityl antibody (clone D11, Cell Signaling Technology) specifically recognizes and can be used to quantify histone H2BK119/K123 ubiquitination in budding as well as fission yeast cells.

In summary, employing a serial dilution of denatured protein lysates, a sectioned blot probing approach, and Ponceau S-stained proteins for loading control normalization provides a highly sensitive and effective means for quantitating histone H2B monoubiquitination in the yeast model systems.

## 5. Reagents Setup

### 5.1. Preparation of Media and Solutions

56. To grow *S. cerevisiae* strains, prepare YPAD medium by dissolving 10 g yeast extract, 20 g bacto-peptone, 40 mg adenine hemisulfate in 950 mL water. Sterilize media by autoclaving at 121 °C for 20 min. Add 50 mL sterile 40% dextrose/glucose solution to the autoclaved YPA media after cooling to room temperature. To grow *S. pombe* strains, dissolve 35.25 g YES50 powder in 1 L water and sterilize by autoclaving. To prepare solid media, add agar (2%) to liquid media, sterilize by autoclaving, and cool before pouring into Petri plates.
57. For washing and lysing yeast cells, prepare 5% and 20% trichloroacetic acid (TCA), respectively, by diluting 6.1 N TCA (or 100% stock solution) in sterile water immediately before use. You will need to prepare 1 mL 5% TCA and 0.5 mL 20% TCA for processing each sample. Calculate the total volume of these working solutions needed based on the number of samples or strains in each experiment.
58. Prepare 1X SDS lysis buffer by diluting the 2X Laemmli sample buffer (Bio-Rad) in sterile water. Do not add reducing agents (β-mercaptoethanol or DTT) to the SDS lysis buffer, as they will interfere with the *DC* protein assay. To neutralize the acidic extracts, prepare 2 M Tris base by dissolving 12.114 g Tris in 50 mL of water and sterilize by filtration.
59. To reconstitute the stock SDS loading buffer, combine 950 μL 2X Laemmli sample buffer (Bio Rad) with 50 μL β-mercaptoethanol. Dilute the reconstituted 2X dye 1:1 with sample. To prepare 6X SDS loading dye, combine 300 mM Tris.HCl, pH 7.5, 12%SDS, 60% glycerol and 0.12% bromophenol blue in water. Heat to liquefy and combine 100 μL 6X SDS loading dye with 6 μL β-mercaptoethanol. Mix this with the sample at 1:6 ratio. For electrophoresis, prepare 10X SDS running buffer by dissolving 15.1 g Tris base, 72 g glycine, and 5 g SDS in 1 L water.
60. For reversible staining of the blots, mix 0.4 g Ponceau S dye, 8 mL TCA, and 2 mL acetic acid in 100 mL water.
61. For probing and washing blots, first prepare 10X PBS by dissolving 17.8 g Na_2_HPO_4_. 2H_2_O, 2.4 g KH_2_PO_4_, 80 g NaCl and 2 g KCl in water, and sterilize by autoclaving. Then, prepare 1 L 1X PBST solution by diluting 100 mL 10X PBS and 5 mL 10% Tween-20 in water. Dissolve 0.5 g non-fat dry milk in 10 mL 1X PBST to prepare 5% blocking solution for the blots. Dilute equal parts of 5% milk stock in 1X PBST to obtain 2.5% milk solution to prepare primary or secondary antibodies solutions. To prepare Towbin transfer buffer, dissolve 57.5 g glycine and 12 g Tris base in water, add 800 mL methanol and 4 mL 10% SDS, and bring to 4 L with water.

### 5.2. Generation of S. cerevisiae Strains

Gene knockouts in budding yeast were created in parental DHY214/DHY217 or their derivatives. The coding region of a target gene was deleted in yeast using PCR products containing the target locus to be disrupted along with the replacement *KanMX6* selection cassette amplified from genomic DNA isolated from the respective deletion mutant in Open Biosystem’s yeast collection. Alternatively, ~40 bp sequences homologous to the promoter and 3′ terminator regions of a target gene were amplified using pF6a-KanMX or pAG25 (natMX4) as the template [36]. PCR products containing ~40 bp sequences homologous to the 3′-end of the open reading frame minus the stop codon and the 3′ terminator regions of *HTB1* and *HTB2* genes (coding for histone H2B) and additionally containing a V5 epitope-tag were amplified using pMC001 as the template (to be described elsewhere). The final single- or double-gene deletion or epitope-tagged strains were created through mating, sporulation, and tetrad dissection. Genotypes of budding and fission yeast strains [37] used in this study are listed in Supplementary Table S1.

## Data Availability

Not applicable.

## Supplementary Materials

The following is available online at https://www.mdpi.com/article/10.3390/mps5050074/s1: Table S1: Genotypes of yeast strains used in this study.
