# Supplementary material for: Quantitative Assessment of Histone H2B Monoubiquitination in Yeast Using Immunoblotting
Source: Methods Protoc. 2022 Sep 24;5(5):74. doi: 10.3390/mps5050074 (PMC9609377; doi:10.3390/mps5050074)
Supplement: Supplementary file 1 [file mps-05-00074-s001.zip › mps-1911869-supplementary.pdf]

Table S1. Genotypes of yeast strains used in this study.

Saccharomyces cerevisiae strains

Strain	Genotype	Source
DHY217	*MATa his3Δ1 leu2Δ0 ura3Δ0*	(a)
YMC181	*MATa his3Δ1 leu2Δ0 ura3Δ0 arg4Δ rad6Δ::KANMX*	(b)
YMC183	*MATa his3Δ1 leu2Δ0 ura3Δ0 arg4Δ bre1Δ::KANMX*	(b)
YMC185	*MATa his3Δ1 leu2Δ0 ura3Δ0 arg4Δ lge1Δ::KANMX*	(b)
YMC191	*MATa his3Δ1 leu2Δ0 ura3Δ0 arg4Δ ubp8Δ::KANMX*	(b)
YMC193	*MATa his3Δ1 leu2Δ0 ura3Δ0 arg4Δ ubp10Δ::NATMX*	(b)
YMC195	*MATa his3Δ1 leu2Δ0 ura3Δ0 arg4Δ ubp8Δ::KANMX ubp10Δ::NATMX*	(b)
YMC203	*MATa his3Δ1 leu2Δ0 ura3Δ0 arg4Δ HTB1 K123R::NATMX HTB2 K123R::KANMX*	(b)
YMC233	*MATa his3Δ1 leu2Δ0 ura3Δ0 arg4Δ HTB1V5-HYGMX-2xFLAG HTB2V5-LEU-2xFLAG*	(b)

Schizosaccharomyces pombe strains

Strain	Genotype	Source
KGY246	*ade6-M210 leu1-32 ura4-D18 h-*	(d)
KGY12992	*ubp8::ura4+ ade6-M210 leu1-32 ura4-D18 h-*	(d)
KGY15109	*htb1-K119R::kanR ade6-M21X leu1-32 ura4-D18 h?*	(d)
KGY15253	*htb1-FLAG:kanR ade6-M21X h-*	(c)
KGY15353	*brl1::kanR ade6-M21X leu1-32 ura4-D18 h+*	(d)
KGY15388	*htb1-K119R-FLAG:kanR ade6-M21X h-*	(c)

a) Kindly provided by Late John Horecka.
b) This study.
c) Tanny, J.C.; Erdjument-Bromage, H.; Tempst, P.; Allis, C.D. *Genes Dev* 2007, *21*, 835-847.
d) Elmore, Z.C.; Beckley, J.R.; Chen, J.S.; Gould, K.L. *G3 (Bethesda)* 2014, *4*, 1529-1538.
